# 3,4-Bis(4-bromo­phen­yl)-*N*-phenyl­maleimide

**DOI:** 10.1107/S1600536811014152

**Published:** 2011-04-22

**Authors:** Yong Liu, Shuai Zheng, Jing Zhou, Dongmei Gao, Zhen-Ting Du

**Affiliations:** a Medical College, Northwest University for Nationalities, Lanzhou 730030, Gansu Province, People’s Republic of China; b College of Science, Northwest A&F University, Yangling 712100, Shannxi Province, People’s Republic of China

## Abstract

In the title mol­ecule, C_22_H_13_Br_2_NO_2_, the three benzene rings are arranged in a propeller-like fashion around the central malemide ring, making dihedral angles of 48.2 (4), 30.2 (4) and 34.8 (4)° with the malemide ring. The C—C single-bond lengths connecting benzene groups and maleimide are significantly shorter [C—C = 1.468 (9) and 1.478 (9) Å] than a typical C*sp* ^3^—C*sp* ^3^ single bond, indicating partial conjugation between the benzene and the mal­eimide. A weak non­classical C—H⋯O hydrogen bond helps to stabilize the crystal structure.

## Related literature

For general background to 3,4-diaryl-substituted maleimide derivatives, see: Fujii *et al.* (2001 ▶); Onimura *et al.* (2010 ▶); Shorunov *et al.* (2006 ▶).
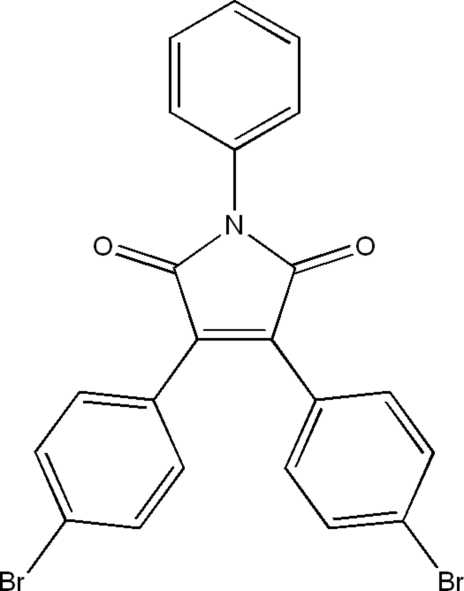


## Experimental

### 

#### Crystal data

C_22_H_13_Br_2_NO_2_
*M* *_r_* = 483.13
Monoclinic, 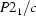

*a* = 10.844 (5) Å
*b* = 18.594 (9) Å
*c* = 9.602 (5) Å
β = 102.760 (6)°
*V* = 1888.3 (16) Å^3^
*Z* = 4
Mo *K*α radiation
μ = 4.31 mm^−1^
*T* = 296 K
0.32 × 0.30 × 0.24 mm

#### Data collection

Bruker APEXII CCD diffractometer
Absorption correction: multi-scan (*SADABS*; Sheldrick, 1998 ▶) *T*_min_ = 0.339, *T*_max_ = 0.424
8594 measured reflections
3382 independent reflections
1427 reflections with *I* > 2σ(*I*)
*R*_int_ = 0.117

#### Refinement

*R*[*F* ^2^ > 2σ(*F* ^2^)] = 0.050
*wR*(*F* ^2^) = 0.133
*S* = 0.94
3382 reflections
244 parameters
H-atom parameters constrained
Δρ_max_ = 0.51 e Å^−3^
Δρ_min_ = −0.71 e Å^−3^

### 

Data collection: *APEX2* (Bruker, 2005 ▶); cell refinement: *SAINT* (Bruker, 2001 ▶); data reduction: *SAINT*; program(s) used to solve structure: *SHELXS97* (Sheldrick, 2008 ▶); program(s) used to refine structure: *SHELXL97* (Sheldrick, 2008 ▶); molecular graphics: *SHELXTL* (Sheldrick, 2008 ▶); software used to prepare material for publication: *SHELXTL*.

## Supplementary Material

Crystal structure: contains datablocks global, I. DOI: 10.1107/S1600536811014152/rk2272sup1.cif

Structure factors: contains datablocks I. DOI: 10.1107/S1600536811014152/rk2272Isup2.hkl

Additional supplementary materials:  crystallographic information; 3D view; checkCIF report

## Figures and Tables

**Table 1:** Hydrogen-bond geometry (Å, °)

*D*—H⋯*A*	*D*—H	H⋯*A*	*D*⋯*A*	*D*—H⋯*A*
C21—H21⋯O1^i^	0.93	2.41	3.267 (9)	153

Symmetry code: (i) 
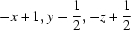
.

**Fig. 1.**
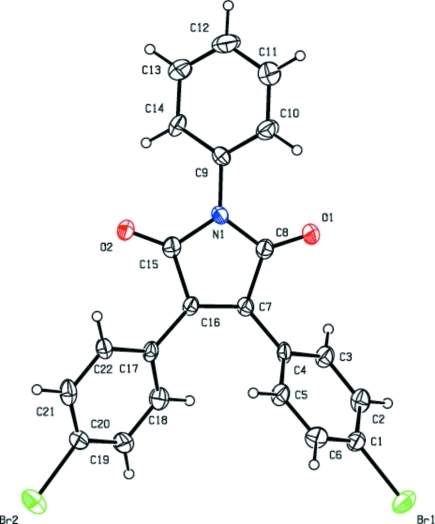
The molecular structure of I with the atom numbering scheme. Displacement ellipsoids are drawn at the 30% probability level. H atoms are presented as a small spheres of arbitrary radius.

**Fig. 2.**
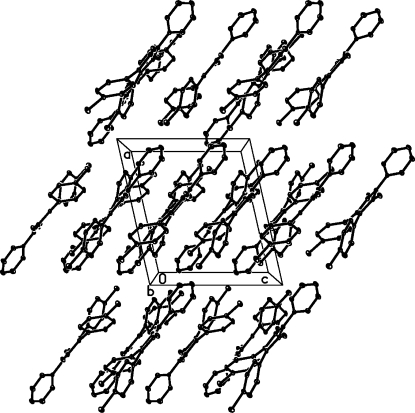
The molecular packing of I along b axis.

## supplementary crystallographic information

### Comment

3,4-Diaryl-substituted maleimide is a conjugated unit which has interesting optical and electronic properties. A number of 3,4-diaryl-substituted maleimide derivatives have been designed and synthesized to be used as monomer of some electro–optic polymers (Shorunov *et al.*, 2006; Onimura *et al.*, 2010). In the course of exploring new electro–optic compounds, we obtained a intermediate compound 3,4-bis(4-bromophenyl)-*N*-phenylmaleimide I. Here we report the structure and synthesis of title compound.

The molecule holds four rings. The maleimide ring locates the core position, and the other three benzene rings are arranged in a propeller–like fashion around the central malemide 5–membering ring with the dihedral angeles being 48.2 (4)° (C1–C6), 30.2 (4)° (C9–C14) and 34.8 (4)° (C17–C22). The C—C single bond lengths conecting benzene groups and maleimide unit are respectively 1.468 (9)Å (C4—C7) and 1.478 (9)Å (C17—C18), which are obviously shorter than typical C*sp*^3^—C*sp*^3^ single bond. This means that the bonding between the benzenes and the maleimide is quite conjugated.

The molecules of I are crystalized in *P*2_1_/*c* space group which is different from that of *N*–3,4–triphenylmaleimide (*Pbca*, Fujii *et al.*, 2001). There are no classic hydrogen bonds in this crystal structure. However, the weak intermolecular interaction C21—H21···O1^i^ is helpful to the stabilization of the packing (Fig. 2). This intermolecular non–classical H–bond is characterized by the parameters: bond lengths of 0.93Å (C21—H21), 2.41Å (H21···O1^i^), 3.267 (9)Å (C21···O1^i^) and angle 153° (C21—H21···O1^i^). Symmetry code: (i) 1-*x*, -1/2+*y*, 1/2-*z*.

### Experimental

3,4–Bis(4–bromophenyl)maleic anhydride (0.60 g, 1.47 mmol), 4–methylbenzenesulfonic acid (0.30 g, 3.26 mmol) and H_2_SO_4_ (2 ml) were dissolved in *N*,*N*–dimethylformamide (*DMF*, 1.0 ml) and toluene (20 ml) mixed solvent. After heating the mixture to refluxing, a toluene solution of anilin (0.20 g, 2.17 mmol) was slowly added. After stirring for 2 h, the mixture was cooled to 333 K and poured into 8% Na_2_CO_3_ solution and further stirred for 10 min. The solution was extracted with toluene and dried over Na_2_SO_4_. After removing the solvent, the crude product was purified by recrystallization from ethanol, affording the title compound, I, (0.51 g, 72%). Then the compound I was dissolved in *THF*, and yellow crystals were formed on slow evaporation at room temperature over one week.

### Refinement

All H atoms were placed in geometrically calculated positions and refined using a riding model with C—H = 0.93%A (for aromatic H) with *U*_iso_(H) = 1.2*U*_eq_(C).

### Crystal data

**Table:**

C_22_H_13_Br_2_NO_2_	*F*(000) = 952
*M**_r_* = 483.13	*D*_x_ = 1.699 Mg m^−^^3^
Monoclinic, *P*2_1_/*c*	Mo *K*α radiation, λ = 0.71073 Å
Hall symbol: -P 2ybc	Cell parameters from 1226 reflections
*a* = 10.844 (5) Å	θ = 2.2–20.8°
*b* = 18.594 (9) Å	µ = 4.31 mm^−^^1^
*c* = 9.602 (5) Å	*T* = 296 K
β = 102.760 (6)°	Block, yellow
*V* = 1888.3 (16) Å^3^	0.32 × 0.30 × 0.24 mm
*Z* = 4	

### Data collection

**Table:**

Bruker APEXII CCD diffractometer	3382 independent reflections
Radiation source: fine–focus sealed tube	1427 reflections with *I* > 2σ(*I*)
graphite	*R*_int_ = 0.117
φ and ω scans	θ_max_ = 25.2°, θ_min_ = 2.2°
Absorption correction: multi-scan (*SADABS*; Sheldrick, 1998)	*h* = −12→12
*T*_min_ = 0.339, *T*_max_ = 0.424	*k* = −22→21
8594 measured reflections	*l* = −9→11

### Refinement

**Table:**

Refinement on *F*^2^	Primary atom site location: structure-invariant direct methods
Least-squares matrix: full	Secondary atom site location: difference Fourier map
*R*[*F*^2^ > 2σ(*F*^2^)] = 0.050	Hydrogen site location: inferred from neighbouring sites
*wR*(*F*^2^) = 0.133	H-atom parameters constrained
*S* = 0.94	*w* = 1/[σ^2^(*F*_o_^2^) + (0.0468*P*)^2^] where *P* = (*F*_o_^2^ + 2*F*_c_^2^)/3
3382 reflections	(Δ/σ)_max_ = 0.001
244 parameters	Δρ_max_ = 0.51 e Å^−^^3^
0 restraints	Δρ_min_ = −0.71 e Å^−^^3^

### Special details

**Table:**

Geometry. All s.u.'s (except the s.u. in the dihedral angle between two l.s. planes) are estimated using the full covariance matrix. The cell s.u.'s are taken into account individually in the estimation of s.u.'s in distances, angles and torsion angles; correlations between s.u.'s in cell parameters are only used when they are defined by crystal symmetry. An approximate (isotropic) treatment of cell s.u.'s is used for estimating s.u.'s involving l.s. planes.
Refinement. Refinement of *F*^2^ against ALL reflections. The weighted *R*–factor *wR* and goodness of fit *S* are based on *F*^2^, conventional *R*–factors *R* are based on *F*, with *F* set to zero fornegative *F*^2^. The threshold expression of *F*^2^ > σ(*F*^2^) is used only for calculating *R*–factors(gt) *etc*. and is not relevant to the choice of reflections for refinement. *R*–factors based on *F*^2^ are statistically about twice as large as those based on *F*, and *R*–factors based on ALL data will be even larger.

### Fractional atomic coordinates and isotropic or equivalent isotropic displacement parameters (Å^2^)

**Table:**

	*x*	*y*	*z*	*U*_iso_*/*U*_eq_	
Br1	0.18239 (9)	1.08051 (4)	−0.16015 (9)	0.0659 (4)	
Br2	0.12170 (9)	0.59744 (5)	−0.09998 (9)	0.0681 (4)	
C1	0.2818 (7)	1.0177 (4)	−0.0272 (8)	0.042 (2)	
C2	0.3009 (7)	1.0324 (4)	0.1166 (8)	0.044 (2)	
H2	0.2661	1.0735	0.1474	0.052*	
C3	0.3718 (7)	0.9859 (4)	0.2152 (7)	0.046 (2)	
H3	0.3854	0.9965	0.3121	0.055*	
C4	0.4223 (7)	0.9244 (3)	0.1720 (7)	0.037 (2)	
C5	0.4019 (7)	0.9103 (4)	0.0241 (7)	0.041 (2)	
H5	0.4353	0.8689	−0.0080	0.050*	
C6	0.3333 (8)	0.9573 (4)	−0.0718 (7)	0.049 (2)	
H6	0.3214	0.9480	−0.1690	0.058*	
C7	0.4950 (7)	0.8740 (4)	0.2765 (7)	0.0317 (19)	
C8	0.5979 (7)	0.8995 (4)	0.3949 (7)	0.037 (2)	
C9	0.7668 (7)	0.8405 (4)	0.5799 (7)	0.0324 (19)	
C10	0.8549 (8)	0.8948 (4)	0.5883 (8)	0.049 (2)	
H10	0.8449	0.9299	0.5176	0.059*	
C11	0.9570 (8)	0.8967 (5)	0.7012 (9)	0.064 (3)	
H11	1.0169	0.9329	0.7067	0.077*	
C12	0.9715 (9)	0.8455 (5)	0.8061 (8)	0.066 (3)	
H12	1.0385	0.8483	0.8852	0.079*	
C13	0.8877 (9)	0.7907 (5)	0.7936 (9)	0.067 (3)	
H13	0.9013	0.7541	0.8612	0.080*	
C14	0.7840 (8)	0.7881 (4)	0.6845 (8)	0.049 (2)	
H14	0.7252	0.7513	0.6802	0.059*	
C15	0.5930 (7)	0.7768 (4)	0.4054 (7)	0.038 (2)	
C16	0.4904 (7)	0.8021 (4)	0.2827 (7)	0.0321 (19)	
C17	0.3999 (8)	0.7505 (4)	0.1988 (7)	0.035 (2)	
C18	0.2750 (8)	0.7690 (4)	0.1499 (8)	0.050 (2)	
H18	0.2470	0.8136	0.1741	0.060*	
C19	0.1900 (8)	0.7226 (4)	0.0653 (7)	0.052 (2)	
H19	0.1053	0.7352	0.0353	0.063*	
C20	0.2331 (8)	0.6571 (4)	0.0260 (7)	0.043 (2)	
C21	0.3549 (8)	0.6361 (4)	0.0806 (7)	0.046 (2)	
H21	0.3816	0.5905	0.0607	0.055*	
C22	0.4388 (7)	0.6829 (4)	0.1659 (7)	0.036 (2)	
H22	0.5221	0.6687	0.2014	0.044*	
N1	0.6588 (6)	0.8394 (3)	0.4625 (6)	0.0341 (16)	
O1	0.6229 (5)	0.9610 (3)	0.4283 (5)	0.0494 (15)	
O2	0.6167 (5)	0.7165 (3)	0.4467 (5)	0.0480 (15)	

### Atomic displacement parameters (Å^2^)

**Table:**

	*U*^11^	*U*^22^	*U*^33^	*U*^12^	*U*^13^	*U*^23^
Br1	0.0834 (8)	0.0516 (6)	0.0525 (6)	0.0141 (5)	−0.0068 (5)	0.0128 (4)
Br2	0.0699 (7)	0.0590 (6)	0.0689 (7)	−0.0182 (5)	0.0015 (5)	−0.0198 (5)
C1	0.056 (6)	0.032 (5)	0.034 (5)	0.004 (4)	0.002 (4)	0.000 (4)
C2	0.058 (6)	0.034 (5)	0.043 (5)	0.007 (4)	0.021 (5)	−0.006 (4)
C3	0.073 (7)	0.030 (5)	0.031 (5)	0.008 (5)	0.005 (4)	−0.006 (4)
C4	0.055 (6)	0.018 (4)	0.037 (5)	−0.003 (4)	0.011 (4)	−0.002 (4)
C5	0.064 (6)	0.027 (5)	0.033 (4)	0.012 (4)	0.011 (4)	−0.008 (4)
C6	0.078 (7)	0.048 (5)	0.019 (4)	0.003 (5)	0.008 (4)	−0.007 (4)
C7	0.043 (6)	0.025 (4)	0.027 (4)	0.002 (4)	0.009 (4)	−0.006 (3)
C8	0.047 (6)	0.031 (5)	0.034 (5)	0.000 (4)	0.009 (4)	0.002 (4)
C9	0.039 (6)	0.030 (4)	0.029 (5)	0.000 (4)	0.010 (4)	−0.011 (4)
C10	0.050 (6)	0.054 (6)	0.041 (5)	0.002 (5)	0.004 (5)	0.014 (4)
C11	0.060 (7)	0.055 (6)	0.072 (6)	−0.007 (5)	0.003 (5)	0.008 (5)
C12	0.060 (7)	0.080 (7)	0.047 (6)	0.011 (6)	−0.012 (5)	0.003 (5)
C13	0.072 (8)	0.064 (7)	0.054 (6)	−0.008 (6)	−0.010 (6)	0.021 (5)
C14	0.056 (7)	0.035 (5)	0.052 (6)	0.003 (4)	0.004 (5)	0.019 (4)
C15	0.055 (6)	0.035 (5)	0.025 (5)	−0.003 (4)	0.014 (4)	0.002 (4)
C16	0.051 (6)	0.023 (4)	0.022 (4)	0.004 (4)	0.007 (4)	−0.001 (3)
C17	0.058 (7)	0.024 (5)	0.023 (4)	−0.001 (4)	0.011 (4)	0.003 (3)
C18	0.054 (7)	0.034 (5)	0.059 (6)	0.008 (5)	0.007 (5)	−0.006 (4)
C19	0.058 (7)	0.038 (5)	0.053 (5)	0.003 (5)	−0.004 (5)	−0.010 (4)
C20	0.049 (7)	0.038 (5)	0.038 (5)	−0.005 (4)	0.000 (4)	−0.003 (4)
C21	0.075 (8)	0.029 (5)	0.041 (5)	−0.006 (5)	0.027 (5)	−0.004 (4)
C22	0.047 (6)	0.027 (5)	0.037 (5)	−0.005 (4)	0.012 (4)	−0.003 (4)
N1	0.039 (4)	0.030 (4)	0.031 (4)	−0.008 (3)	0.004 (3)	−0.001 (3)
O1	0.067 (4)	0.028 (3)	0.046 (3)	−0.003 (3)	−0.005 (3)	−0.005 (3)
O2	0.066 (4)	0.028 (3)	0.043 (3)	0.000 (3)	−0.004 (3)	0.004 (2)

### Geometric parameters (Å, °)

**Table:**

Br1—C1	1.883 (7)	C11—C12	1.369 (10)
Br2—C20	1.873 (7)	C11—H11	0.9300
C1—C6	1.365 (9)	C12—C13	1.352 (11)
C1—C2	1.378 (8)	C12—H12	0.9300
C2—C3	1.384 (9)	C13—C14	1.357 (9)
C2—H2	0.9300	C13—H13	0.9300
C3—C4	1.371 (9)	C14—H14	0.9300
C3—H3	0.9300	C15—O2	1.198 (7)
C4—C5	1.414 (8)	C15—N1	1.412 (8)
C4—C7	1.468 (9)	C15—C16	1.506 (9)
C5—C6	1.365 (9)	C16—C17	1.478 (9)
C5—H5	0.9300	C17—C18	1.375 (10)
C6—H6	0.9300	C17—C22	1.385 (9)
C7—C16	1.339 (9)	C18—C19	1.387 (9)
C7—C8	1.484 (9)	C18—H18	0.9300
C8—O1	1.202 (7)	C19—C20	1.386 (9)
C8—N1	1.385 (8)	C19—H19	0.9300
C9—C10	1.380 (9)	C20—C21	1.366 (10)
C9—C14	1.383 (8)	C21—C22	1.388 (9)
C9—N1	1.435 (8)	C21—H21	0.9300
C10—C11	1.369 (10)	C22—H22	0.9300
C10—H10	0.9300		
			
C6—C1—C2	119.7 (6)	C11—C12—H12	120.3
C6—C1—Br1	120.7 (6)	C12—C13—C14	121.4 (8)
C2—C1—Br1	119.6 (6)	C12—C13—H13	119.3
C1—C2—C3	120.0 (7)	C14—C13—H13	119.3
C1—C2—H2	120.0	C13—C14—C9	119.4 (8)
C3—C2—H2	120.0	C13—C14—H14	120.3
C4—C3—C2	120.9 (7)	C9—C14—H14	120.3
C4—C3—H3	119.6	O2—C15—N1	126.1 (7)
C2—C3—H3	119.6	O2—C15—C16	128.3 (7)
C3—C4—C5	118.3 (6)	N1—C15—C16	105.6 (6)
C3—C4—C7	121.0 (6)	C7—C16—C17	130.6 (7)
C5—C4—C7	120.6 (6)	C7—C16—C15	108.6 (6)
C6—C5—C4	120.0 (7)	C17—C16—C15	120.7 (6)
C6—C5—H5	120.0	C18—C17—C22	118.5 (7)
C4—C5—H5	120.0	C18—C17—C16	120.6 (7)
C5—C6—C1	121.1 (7)	C22—C17—C16	121.0 (7)
C5—C6—H6	119.5	C17—C18—C19	121.4 (7)
C1—C6—H6	119.5	C17—C18—H18	119.3
C16—C7—C4	130.3 (6)	C19—C18—H18	119.3
C16—C7—C8	108.3 (6)	C18—C19—C20	119.0 (8)
C4—C7—C8	121.3 (6)	C18—C19—H19	120.5
O1—C8—N1	125.9 (7)	C20—C19—H19	120.5
O1—C8—C7	126.6 (7)	C21—C20—C19	120.1 (7)
N1—C8—C7	107.5 (6)	C21—C20—Br2	120.7 (6)
C10—C9—C14	119.5 (7)	C19—C20—Br2	119.2 (6)
C10—C9—N1	119.4 (6)	C20—C21—C22	120.0 (7)
C14—C9—N1	121.1 (7)	C20—C21—H21	120.0
C11—C10—C9	119.6 (7)	C22—C21—H21	120.0
C11—C10—H10	120.2	C17—C22—C21	120.6 (7)
C9—C10—H10	120.2	C17—C22—H22	119.7
C10—C11—C12	120.4 (9)	C21—C22—H22	119.7
C10—C11—H11	119.8	C8—N1—C15	109.6 (6)
C12—C11—H11	119.8	C8—N1—C9	125.3 (6)
C13—C12—C11	119.5 (8)	C15—N1—C9	124.9 (6)
C13—C12—H12	120.3		
			
C6—C1—C2—C3	−0.1 (12)	O2—C15—C16—C7	178.1 (8)
Br1—C1—C2—C3	178.8 (6)	N1—C15—C16—C7	−2.8 (8)
C1—C2—C3—C4	−1.0 (12)	O2—C15—C16—C17	2.3 (12)
C2—C3—C4—C5	1.0 (11)	N1—C15—C16—C17	−178.6 (6)
C2—C3—C4—C7	−178.5 (7)	C7—C16—C17—C18	−32.3 (12)
C3—C4—C5—C6	−0.1 (11)	C15—C16—C17—C18	142.5 (7)
C7—C4—C5—C6	179.5 (7)	C7—C16—C17—C22	146.8 (8)
C4—C5—C6—C1	−1.0 (12)	C15—C16—C17—C22	−38.4 (10)
C2—C1—C6—C5	1.1 (12)	C22—C17—C18—C19	−2.3 (11)
Br1—C1—C6—C5	−177.8 (6)	C16—C17—C18—C19	176.9 (7)
C3—C4—C7—C16	135.2 (9)	C17—C18—C19—C20	−1.9 (12)
C5—C4—C7—C16	−44.3 (12)	C18—C19—C20—C21	5.8 (12)
C3—C4—C7—C8	−48.7 (10)	C18—C19—C20—Br2	−175.2 (6)
C5—C4—C7—C8	131.7 (7)	C19—C20—C21—C22	−5.5 (12)
C16—C7—C8—O1	−174.1 (8)	Br2—C20—C21—C22	175.5 (5)
C4—C7—C8—O1	9.1 (12)	C18—C17—C22—C21	2.7 (10)
C16—C7—C8—N1	4.7 (8)	C16—C17—C22—C21	−176.5 (6)
C4—C7—C8—N1	−172.1 (6)	C20—C21—C22—C17	1.2 (11)
C14—C9—C10—C11	−1.1 (11)	O1—C8—N1—C15	172.3 (7)
N1—C9—C10—C11	179.0 (7)	C7—C8—N1—C15	−6.5 (8)
C9—C10—C11—C12	−0.5 (13)	O1—C8—N1—C9	−3.4 (12)
C10—C11—C12—C13	3.5 (14)	C7—C8—N1—C9	177.8 (6)
C11—C12—C13—C14	−4.8 (15)	O2—C15—N1—C8	−175.1 (7)
C12—C13—C14—C9	3.2 (14)	C16—C15—N1—C8	5.8 (8)
C10—C9—C14—C13	−0.2 (11)	O2—C15—N1—C9	0.6 (12)
N1—C9—C14—C13	179.7 (7)	C16—C15—N1—C9	−178.5 (6)
C4—C7—C16—C17	−9.3 (14)	C10—C9—N1—C8	−33.2 (10)
C8—C7—C16—C17	174.2 (7)	C14—C9—N1—C8	146.9 (7)
C4—C7—C16—C15	175.3 (7)	C10—C9—N1—C15	151.7 (7)
C8—C7—C16—C15	−1.1 (8)	C14—C9—N1—C15	−28.2 (10)

### Hydrogen-bond geometry (Å, °)

**Table:**

*D*—H···*A*	*D*—H	H···*A*	*D*···*A*	*D*—H···*A*
C21—H21···O1^i^	0.93	2.41	3.267 (9)	153

Symmetry codes: (i) −*x*+1, *y*−1/2, −*z*+1/2.
